# Assessment of choriocapillary blood flow changes in response to half-dose photodynamic therapy in chronic central serous chorioretinopathy using optical coherence tomography angiography

**DOI:** 10.1186/s12886-020-01674-9

**Published:** 2020-10-07

**Authors:** Juejun Liu, Changzheng Chen, Lu Li, Yishuang Xu, Zuohuizi Yi, Lu He, Hongmei Zheng

**Affiliations:** Eye Center, Renmin Hospital of Wuhan University, Wuhan, China

**Keywords:** Chronic, Central serous Chorioretinopathy, Optical coherence tomography angiography, Photodynamic therapy

## Abstract

**Background:**

Optical coherence tomography angiography (OCTA) is a newly developed imaging quantitative technique for analysis of choriocapillaris (CC) flow changes, thereby exploring the pathological mechanism of chronic central serous chorioretinopathy (CCSC) and the therapeutic effects of photodynamic therapy (PDT). In this study, we sought to quantify the blood flow changes in CC of CCSC patients receiving half-dose PDT using OCTA.

**Methods:**

A total of 28 affected eyes and 24 unaffected eyes of 26 CCSC patients receiving half-dose PDT, and 40 eyes of 20 healthy gender- and age-matched subjects were retrospectively enrolled in this study. The proportion of total areas of flow signal voids (FSV, %) in CC level of OCTA was assessed in both eyes of the CCSC patients at baseline and repeated in multiple sections at 1-week, 1-month, 3-month and 6-month intervals after PDT. In addition, the CC patterns in response to PDT at early stage and the subsequent morphologic changes were qualitatively documented using OCTA.

**Results:**

For affected eyes, FSV at 6-m follow-up was significantly lower than that at 1-m follow-up (*p* = 0.036). When compared to normal control eyes, FSV in affected eyes was significantly higher at 1-m, 3-m and 6-m follow-up (*p* < 0.05 for all), and FSV in unaffected eyes was significantly higher at baseline, 1-w, 1-m and 3-m follow-up (*p* < 0.05 for all). Three CC patterns of early response to PDT were identified, including signs of recovery with more even flow signals, transient appearance of worse ischemia and secondary neovascularization within CC level.

**Conclusion:**

Abnormal CC flow attenuation remains in completely resolved eyes of CCSC patients treated with half-dose PDT.

## Background

With the development of fundus imaging technology, the potential mechanism of choroidal hemodynamic changes in central serous chorioretinopathy (CSC) has been elucidated using indocyanine green angiography (ICGA) and enhanced depth imaging optical coherence tomography (EDI-OCT) [1]. Choriocapillaris (CC) ischemia processes, along with increasing hydrostatic pressure from choroidal pachyvessels, could result in focal or defused retinal pigment epithelium (RPE) barrier breakdown and subsequent serous retinal detachment (SRD) [1–3]. It has been reported that photodynamic therapy (PDT), with modified parameters, has a long-term efficacy and safety profile in chronic CSC (CCSC) therapy, with fewer complications and greater improvements in vision, subretinal fluid (SRF) and subfoveal choroidal thickness (SFCT) [1, 4]. As demonstrated by previous pathological and angiographic studies, PDT can cause short-term CC hypoperfusion and long-term choroidal vascular remodeling in the treatment of CSC [5]. Assessment of CC in vivo could greatly help understand the pathological mechanism of CSC and the therapeutic effects of PDT [6, 7].

Optical coherence tomography angiography (OCTA) is a newly developed imaging technique, which provides new insights into CC perfusion and vasculature patterns over the selected layers free from dye injection, and greatly compensates for the deficiencies of OCT and ICGA in the observation of CC [7, 8]. Several OCTA-related studies reported irregular flow patterns of CC in patients with CSC, including CC dilatation with increasing flow signals [9, 10], and focal or defused dark areas with flow signal voids (FSV) [7, 9], which were associated with the changes in ICGA. Various binary quantifications of OCTA imaging with objective data on various parameters further confirmed the attenuation of CC in CSC [8, 11, 12]. Previously, we have qualitatively documented that 97% (32/33) of eyes diagnosed with CCSC returned to exhibiting relatively normal distribution of fine particles in CC layer of OCTA at 3-m follow-up after half-dose PDT [13]. Nassisi M, et, al. reported that the CC vessel density at 1-m follow-up was even higher than the baseline value analyzed using an alternative binary approach [14], which however did not exclude the shadowing artifacts of SRF at enrollment. This present study was designed to assess CC blood flow changes in the affected eyes of CCSC patients in response to half-dose PDT assessed at early and long-term follow-ups using OCTA. The results were compared with those of the contralateral unaffected eyes as well as the healthy control eyes to explore the possible differences between them in response to half-dose PDT.

## Methods

Patients with CCSC and healthy gender- and age-matched subjects, from November 2017 to November 2018, were retrospectively enrolled in this study, and all participants provided written informed consent. CCSC is defined [10] as exhibition of visual acuity symptoms for at least 6 months along with documented clinical features of CSC, including macular SRF, RPE changes of leakage and hyperfluorescent alterations detected by OCT and FFA/ICGA. All patients in this study were subjected to half-dose PDT with verteporfin infusion (3 mg/m^2^ of body surface area), and the laser light (689 nm; 50 J/cm^2^ for 83 s) was projected at the area of hyperpermeability in ICGA for 15 min after the start of verteporfin infusion. PDT was performed by an experienced ophthalmologist. The patients with eyes subjected to any previous treatment or exhibiting myopia more than 3.0 diopters, as well as those suffering from other diseases like PCV and neovascular maculopathy (eg, age-related macular degeneration, pathologic myopia, and idiopathic CNV) were excluded from the study upon clinical examination.

All patients had received thorough ophthalmic examinations prior to PDT treatment, including best-corrected visual acuity (BCVA) assessment, fundus fluorescein angiography (FFA), ICGA (HRA2; Heidelberg Engineering, Heidelberg, Germany), fundus photography (Zeiss, Oberkochen, Germany), spectral-domain OCT (SD-OCT) and OCTA (RTVue XR AngioVue Version 2017.1; Optovue Inc., Fremont, CA, USA). Quantitative assessments were performed at baseline and repeated in multiple sections at 1-week, 1-month, and 3-month and 6-month intervals after PDT treatment. These assessments included central macular thickness (CMT) in the mode of retina map of SD-OCT, subfoveal choroidal thickness (SFCT) in the mode of Enhanced HD Line (Enhanced HD-OCT) of SD-OCT, and the proportion of total areas of FSV (%) in OCTA using binary processing. Healthy gender- and age-matched subjects without ocular diseases were enrolled at a single visit and examinations of indirect ophthalmoscopy, BCVA, SD-OCT and OCTA were performed. Of those healthy subjects, those with systemic diseases like diabetes, hypertension, myopia more than 3.0 diopters and ocular media opacity that could affect the scan quality of OCTA were excluded from the study.

**Fig. 1:**
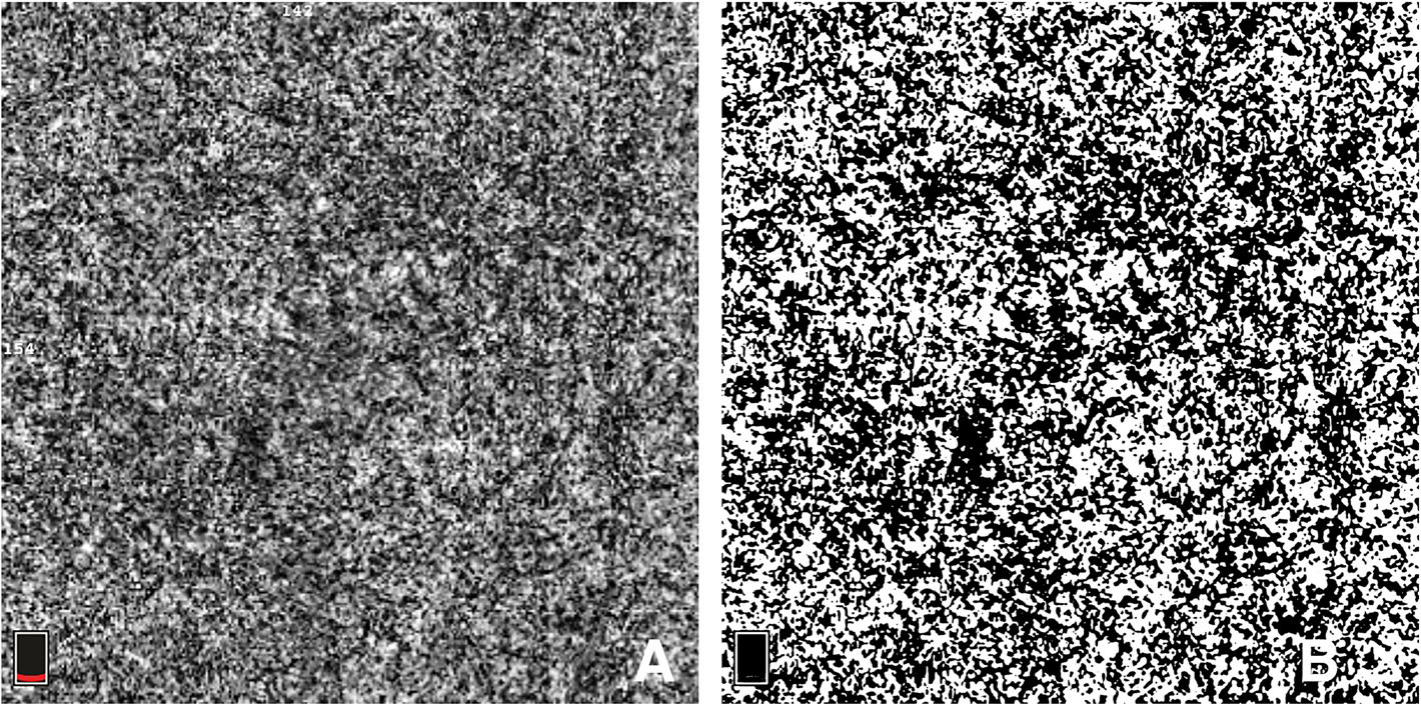
The diagrammatic sketch of automatic quantitative binarization using ImageJ (**b**) of original image of en-face OCTA image of CC slab (**a**)

Enhanced HD-OCT was performed automatically focusing on the macular fovea with a horizontal single-line scanning, the scanning was repeated more than twice and the best one (the clearest image with a signal strength index of more than 70% and without eye movement artifacts) was selected. SFCT is defined as the vertical distance between the outside boundary of RPE and the choroidal-scleral interface determined by averaging the values obtained at three positions: the subfovea area, 750 μm nasal and 750 μm temporal to the fovea. OCTA, using a light source of 840 nm and an A-scan rate of 70,000 scans per second, was incorporated with split-spectrum amplitude-decorrelation angiography (SSADA) algorithm to extract angiographic information by quantifying the decorrelation of the OCT reflectance between the two consecutive B-scans, each containing 304 A-scans at each location on the retina [15]. 3 × 3 mm^2^ OCTA scanning was acquired centering on the fovea with eye tracking option setting on. Multiple scans (more than twice) were performed and images with quality score greater than or equal to 7/10 and without eye movement artifacts were preserved. The CC level is automatically defined as the distance between 10 μm of the upper-bruch’s membrane and 10 μm of the lower-bruch’s membrane. Manual adjustment was performed to ensure the scan was focused on the center of the macula and correct inaccurate automated segmentation. By referring to the reproducible automatic binarization methods reported previously [8, 11], we measured the percentage of black pixels representing absent or decreased flow signal within CC, known as FSV (%) [8, 11], using image J software (ImageJ 1.52a, https://imagej.nih.gov/ij/ docs/) (Fig. 1).

All statistical tests were performed using SPSS version 23.0 (https://www.ibm.com/a nalytics /spss-statistics-software). Categorized data are presented as frequency and percentage, and quantitative data are presented as mean ± standard deviation. Shapiro-Wilk test was used to evaluate distribution. Student’s t-test and one-way analysis of variance (ANOVA) were used for comparing variables between groups. Differences in proportions of gender were analyzed by *X*^2^ [2] test. Pearson or Spearman method was used to analyze the correlation between FSV and age, and between FSV and SFCT. A *P*-value < 0.05 was considered statistically significant.

## Results

**Fig. 2:**
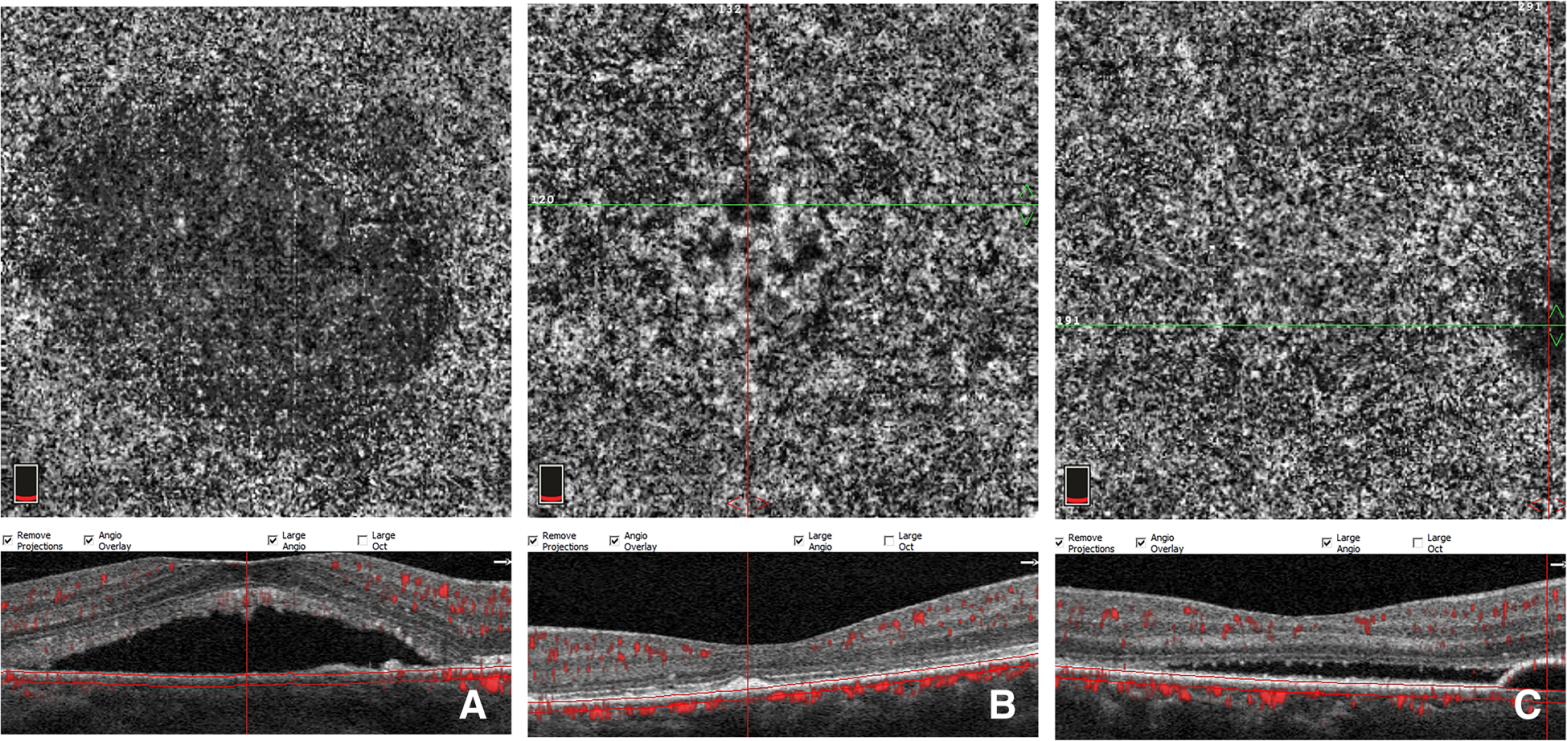
En-face OCTA image of CC slab in affected eyes showing false positive FSV from subfoveal retinal detachment (**a**), RPE clumping (**b**) and PED (**c**) on the corresponding cross-sectional B-scan OCT.

A total of 28 affected eyes and 24 unaffected eyes from 26 CCSC patients, and 40 eyes of 20 age- and gender-matched healthy individuals were enrolled in this study. The baseline characteristics of the CCSC patients were as follows: 19 (73.08%) males and 7 (26.92%) females with an average age of 45.14 ± 7.05 years, and there were no statistically significant differences in age and gender between healthy individuals and CCSC patients (*p* = 0.084, *p* = 0.899, respectively). OCTA imaging with SRF, pigment epithelial detachment (PED), or RPE clumping was excluded from quantitative analysis of FSV (Fig. 2).

FSV Measurement was performed on both eyes of CCSC patients with complete resolution of SRF during follow-ups (Table 1). At 6-m follow-up, 2 affected eyes showed signs of reoccurred SRF and the patients complained of visual problems, while 7 affected eyes and 5 unaffected eyes were lost to follow-up. Significant improvement of BCVA was found at the last follow-up visit (0.045 ± 0.074 LogMAR) compared to the initial (0.235 ± 0.137 LogMAR) assessment (*p* < 0.001).

**Table 1:** OCT and OCTA parameters of FSV and SFCT in patients with CCSC and healthy individuals

FSV	normal subjects (mean ± SD) %	CCSC eyes (mean ± SD)%		*p*-value		
		Affected eyes	Unaffected eyes	P^a^	P^b^	P^c^
0 M	51.56 ± 3.13% (*n* = 40eyes)	–	53.63 ± 3.09% (*n* = 24eyes)	–	0.020	–
1 M	51.56 ± 3.13%	55.98 ± 3.82% (*n* = 25eyes)	53.69 ± 2.67% (n = 24eyes)	< 0.001	0.010	0.005
3 M	51.56 ± 3.13%	54.75 ± 2.93% (*n* = 27eyes)	53.41 ± 2.38% (n = 24eyes)	< 0.001	0.025	0.042
6 M	51.56 ± 3.13%	53.56 ± 3.28% (*n* = 19eyes)	52.80 ± 2.13% (n = 19eyes)	0.035	0.098	0.758
SFCT	normal subjects (mean ± SD)μm	CCSC eyes (mean ± SD)μm		p-value		
		Affected eyes	Unaffected eyes	P^a^	P^b^	P^c^
0 M	267.73 ± 99.18 (n = 40eyes)	447.29 ± 82.28 (*n* = 28eyes)	397.33 ± 70.57 (n = 24eyes)	< 0.001	< 0.001	0.004
1 M	267.73 ± 99.18	387.50 ± 93.77 (n = 28eyes)	370.62 ± 74.63 (n = 24eyes)	< 0.001	< 0.001	0.484
3 M	267.73 ± 99.18	375.30 ± 95.45 (n = 28eyes)	356.46 ± 73.06 (n = 24eyes)	< 0.001	< 0.001	0.407
6 M	267.73 ± 99.18	375.76 ± 104.15 (*n* = 21eyes)	359.33 ± 69.66 (n = 19eyes)	< 0.001	< 0.001	0.494

*CCSC* chronic central serous chorioretinopathy, *SFCT* subretinal foveal choroidal thickness (μm); FSV (%), proportion of total areas of flow signal voids; P^a^, *p*-values for healthy subjects versus affected eyes using Mann–Whitney U test; P^b^, p-values for healthy eyes versus unaffected eyes using Mann–Whitney U test; P^c^, p-values for affected eyes versus unaffected eyes using Wilcoxon test

The FSV values of affected eyes were greater at 1-m, 3-m and 6-m follow-ups (*p* < 0.05 for all), and the FSV values of unaffected eyes were significantly greater at baseline, 1-w, 1-m and 3-m follow-ups (p < 0.05 for all) than normal control eyes (Table 1). The FSV values in affected eyes were greater than those in unaffected eyes at 1-m and 3-m post-treatment (Table 1). The Wilcoxon test results showed that the FSV value of affected eyes at 6-m follow-up was significantly greater than that at 1-m follow-up (*p* = 0.036). The nonparametric Kruskal-Wallis test results showed that the FSV values of unaffected eyes showed no significant differences between all subgroups of follow-ups (0-m, 1-w, 1-m, 3-m, and 6-m) (*p* = 0.674).

The SFCT values of both eyes of CCSC patients were greater than normal control eyes throughout the follow-up period in this cohort (*p* < 0.05 for all), and significant difference was found between the affected and unaffected eyes of CCSC patients at baseline (*p* = 0.015) (Table 1). Nonparametric Kruskal-Wallis test results showed that the SFCT values of affected eyes in all subgroups (0-m, 1-w, 1-m, 3-m, and 6-m) were positive (*p* = 0.007), and the Post-hoc multiple comparisons within subgroups showed a significant difference in SFCT values between baseline and 3-m follow-up (p = 0.015). However, no statistically significant differences were observed in SFCT between all subgroups (0-m, 1-w, 1-m, 3-m, and 6-m) of the unaffected eyes (*p* = 0.196) using nonparametric Kruskal-Wallis test.

**Fig. 3:**
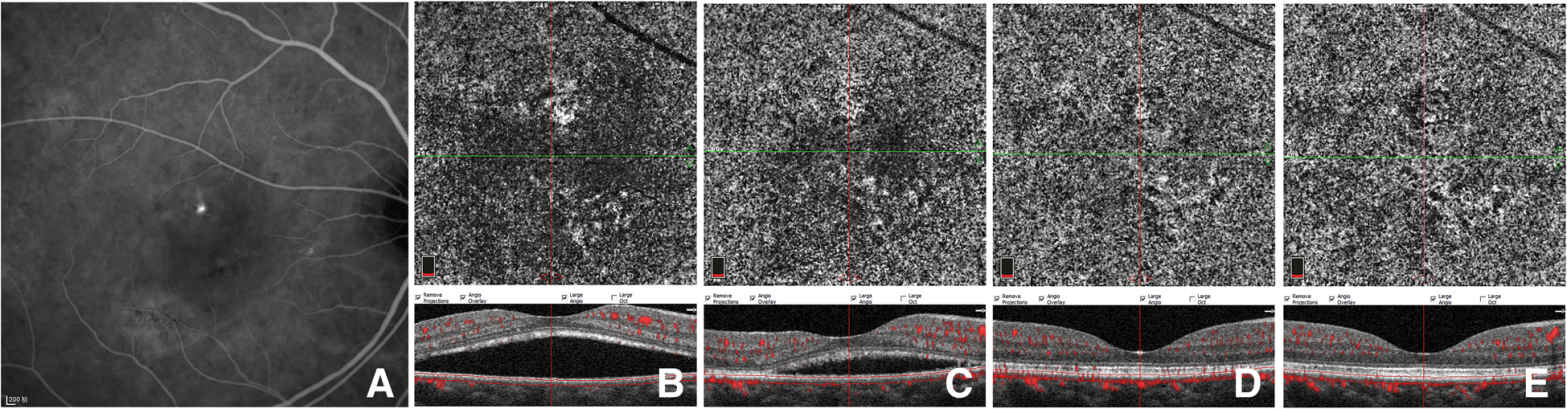
Images of a case showing CC pattern of gradually increasing flow signals after PDT. ICGA (**a**) of baseline reveals partial choriocapillary hyperpermeability (red arrow) and focal hypo-fluorescent areas (blue arrow). En-face OCTA (B-F) of CC slab and the corresponding cross-sectional B-scan OCT (**b**-**f**) demonstrated the CC flow changes with time. Dilatation of CC (red arrow) accompanied by dark areas (blue arrow) can be seen at baseline (**b**). Recovery sign of increasing flow signals and decreasing dark areas was found at 1 week (**c**) after half-dose PDT and at the following 1-m (**d**), 3-m (**e**) and 6-m (**f**) follow-ups, while foci of dark areas (blue arrows) remained

**Fig. 4:**
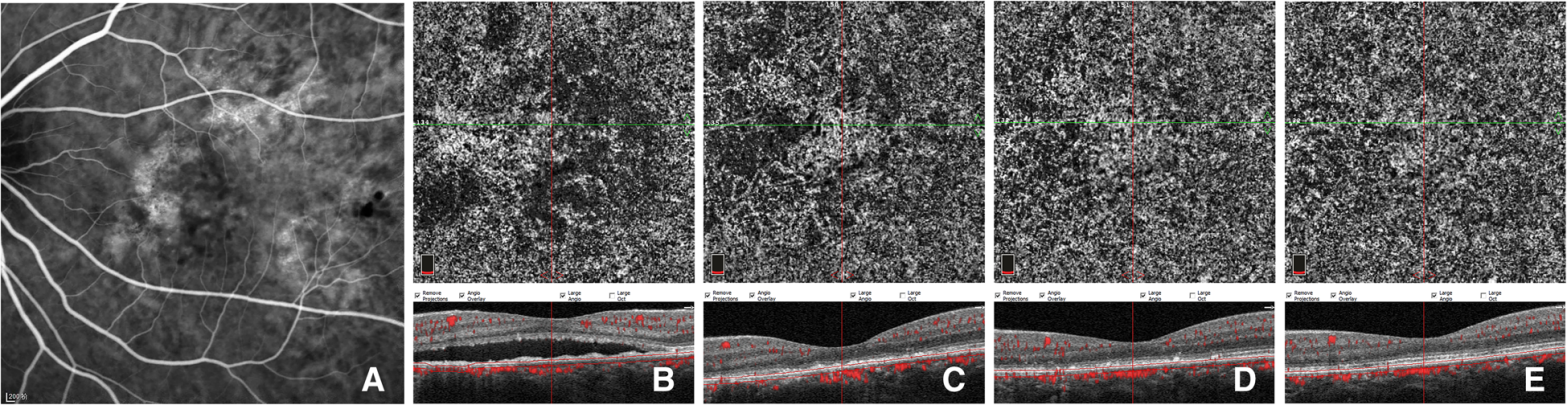
Images of a case showing CC pattern of transient network of neovascularization. ICGA (**a**) of baseline revealed widespread lesions of choriocapillary hyperpermeability (red arrow) with hypo-fluorescent areas within them. En-face OCTA (**b**-**f**) of CC slab and the corresponding cross-sectional B-scan OCT (**b**-**f**) demonstrated the CC flow changes with time. Local dialed CC patterns in macular region surrounded by defused flow signal void were noticeable at baseline (**b**). An emerging network of neovascularization (green arrow) accompanying foci of reduced flow signals (blue arrow) was observed at 1 week after half-dose PDT (**c**), which gradually subsided (green arrows) during subsequent follow-ups of 1 month (**d**), 3 months (**e**) and 6 months (**f**) while focally recovering with CC perfusion (blue arrow)

**Fig. 5:**
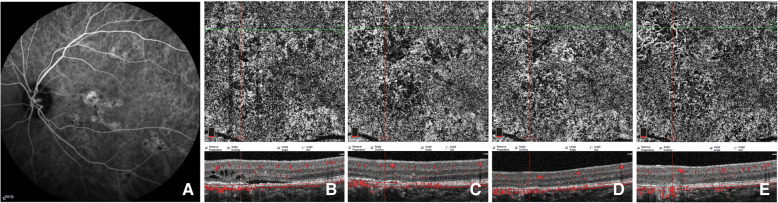
Images of a case showing CC pattern of worse CC ischemia followed by persistent type I CNV. ICGA (**a**) of baseline revealed multifocal choriocapillary hyperpermeability (red arrows). En-face OCTA (**b**-**f**) of CC slab and the corresponding cross-sectional B-scan OCT (**b**-**f**) demonstrated the CC flow changes with time. Defused dilatation of CC (red arrow) (**b**) can be detected at baseline, with punctate dark areas within the lesions. Local worse CC ischemia (blue arrows) was found at 1 week after half-dose PDT (**c**), combined with dynamic changes of neovascularization of sprouts (**d**) at 1-m follow-up, and grew with loose network of CNV (green arrows) during follow-ups of 3 months (**e**) and 6 months (**f**) while focally recovering with CC perfusion (blue arrow)

Due to the inevitable shadowing artifacts of SRF, the FSV values at baseline and early stage after PDT were not quantified. Qualitative observation of CC patterns, as a supplement, showed that all the affected eyes demonstrated local or diffused flow signal attenuation, and dilation and tortuosity of CC at baseline. One week after PDT, three CC patterns of early response to PDT were identified by OCTA. Signs of recovery with increased blood flow signals and decreased dark areas were observed in 21 (75.0%) eyes (Fig. 3), while worsening local CC ischemia was observed in 6 (21.4%) eyes (Fig. 5), and an extraordinarily dilated choriocapillary network or emerging dense network of neovascularization surrounded by foci of reduced flow signals within CC slab was observed in 1 (3.6%) eye (Fig. 4).

Spearman correlation analysis showed that the mean value of FSV was positively correlated with age in the unaffected eyes of CCSC patients at onset (r = 0.469, *p* = 0.021), 1-m (r = 0.470, p = 0.021) and 3-m follow-up (r = 0.414, *p* = 0.044) intervals and also in the eyes of healthy individuals (r = 0.715, *p* < 0.0001). However, no significant correlation was found between the values of FSV and SFCT.

## Discussion

Using OCTA, quantitative analysis with automatic binarization method has been increasingly employed to analyze CC flow changes in some chorioretinal disorders, such as dry age-related macular degeneration and CSC, providing reproducible and objective data [7, 8, 11]. Excluding the masking artifacts of SRF and RPE clumping, PED could help increase the reliability of the quantitative assessment in resolved CSC [2, 11]. In previous studies, assessment of CC flow alterations in affected eyes showed a recovery after PDT [7, 13, 14, 16], which is consistent with our results of the decreased FSV at 6-m follow-up compared to that at 1-m follow-up. However, in this study, the FSA in affected eyes retained higher at 6-m follow-up compared with healthy controls, which indicated that CC attenuation might remain long after the treatment of half-dose PDT, contrary to previous OCTA studies which showed that CC flow returned to normal after half-dose PDT treatment [13, 14, 16]. A series of other follow-up investigations showed that reduced SFCT on EDI-OCT in affected eyes remained higher than healthy controls [1], and choroidal vascular hypermeability on ICGA persisted in some cases even after the SRF had been resolved completely [17, 18]. The remaining choroidopathy observed after half-dose PDT could potentially be attributed to the primary pathogenesis of CSC and/or the therapeutic effects of PDT.

It has been postulated that CSC may be an ocular condition caused by a systemic disease that involves choroidal circulation [1], which could be potentially supported by previous results of some systemic risk factors [1] and abnormalities of CC and SFCT in self-resolved CSC eyes and unaffected fellow eyes [11, 19–21]. Similarly, our study demonstrated that the FSV values of unaffected eyes of CCSC were higher than those in healthy individuals, and remained unchanged over time. As proposed by Nicolo, M [19],. these CC alterations in asymptomatic eyes might be in the early stage of the same condition of CSC [2, 11, 19]. Previous studies also detected punctated hyperfluorescent spots using ICGA in most contralateral eyes of CSC and PCV, which might be a subclinical manifestation of increased choroidal hypermeability and intrachoroidal hydrostatic pressure [17, 22]. And these zones on OCTA with reduced CC flow have been found to be anatomically correlated with pathologically dilated Haller layer vessels on EDI-OCT. [2] In our study, the FSV values of affected eyes were significantly higher at 1-m and 3-m follow-ups, but not at 6-m follow-up than unaffected eyes. We speculated that the abnormal vascular situation in the CC layer in affected eyes of CCSC might recover to a subclinical condition equivalent to that of the contralateral unaffected eyes, which needs to be validated by longitudinal investigations with larger sample size.

In order to evaluate the early CC alterations in response to half-dose PDT in CCSC, qualitative observation was performed and three CC patterns at one week after PDT were documented in the present study. Among the affected eyes, 75.0% (21/28) showed recovering signs of increased flow signals and decreased dark areas (Fig. 3), while 21.4% (6/28) exhibited worse CC ischemia (Fig. 5), which we have previously documented as transient CC ischemia in another cohort [13]. Demircan et al. [16] showed that the transient CC ischemia may occur as early as three days after half-dose PDT. Furthermore, 3.6% (1/28) of the affected eyes demonstrated transient appearance of exuberant neovascularization network within CC level (Fig. 4). Using OCTA, the direct action of PDT on the CC occlusion could be visualized in vivo during follow-up [13, 14, 16]. Post-treatment choroidal hypoperfusion was largely reported with evidence of hypoperfusion on traditional angiography [23] in the treatment of neovascular age-related macular degeneration with full PDT, which might be related to the preferential aggregation of verteporfin in the lesions [23].

The repair mechanisms of surviving endothelial cells and the recanalization processes of novel channels within previously occluded capillaries after PDT treatment remain unclear [1]. In addition to the possibility that CC flow recovers from the released pressure of decreased SFCT owing to the therapeutic effect of PDT [2, 11], it can also be speculated that the damaged choroidal endothelial cells and RPE cells in PDT-treated areas may contribute to the release of VEGF [24, 25], and an imbalanced stimulatory and inhibitory condition for neovascularization formation could be compromised by PDT-related hypoxia and ischemia [25, 26]. However, the process of recanalization could, to some extent, contribute to the formation of CNV [26]. Particularly, two eyes with early CC ischemia, in this cohort, exhibited transient (Fig. 4) and persistent (Fig. 5) appearance of type I CNV, consisting with the morphologic characteristics of neovascularization networks within CC level in previous OCTA-related studies [20, 27, 28]. However, it is controversial that these suspected secondary CNV within CC level may contribute to the CC atrophy and the anterior displacement of medium-sized choroidal vessels with segmentation artifacts that masquerade as CNV [24]. Moreover, type I CNV has been well documented as the most common subtype of secondary CNV in the natural course of CSC [1, 6, 29], which should also be taken into account while studying PDT-related CNV [30]. What makes it more controversial is that most of the previous studies of CSC related CNV were based on patients with heterogeneous treatment histories of PDT or laser photocoagulation [20, 28–30]. Longitudinal OCTA observation would thus help to comparatively follow these lesions to better understand how they behave.

There are some limitations in our study including its retrospective nature, the relatively small number of subjects. Considering the shortage of quantitative method of binarization that limited our ability to assess FSV in affected eyes at the time point of pre-PDT and at the early stage of post-PDT owing to the shadowing artifacts of unresolved subretinal fluid [7], we only compared the FSV values between the time points of 1 m and 6 m after PDT using Wilcoxon test. We ought to record the change terms of exact number of lines or letters in BCVA in this study, which may be more clinically relevant. Our results may underestimate the occurrence of these transient alterations of ischemia and neovascularization occurred within two intervals or outside the imaging area of OCTA. We believe that the actual value of FSV may be higher since patients with SRF were excluded in quantitative assessment, and the choriocapillaris in these patients was certainly abnormal. Although the FSV value was positively correlated with age in healthy controls in consistence with aging physiological changes of CC [8, 11], the dynamic nature of CC flow changes with time should also be taken into consideration in analysis. In addition, the correlation between SFCT and FSV has not been established in this cohort, nor in other reports [31]. It might be due to the relatively small sample size, or that SFCT generally assesses choroidal thickness and both pathologically dilated vessels and increased stromal contribute to the increase of SFCT. Future studies should further evaluate the correlation of FSV with other more specific indicators such as choroidal vascularity index (CVI) [3].

## Conclusions

In summary, we observed that abnormal CC flow attenuation remained in clinically resolved eyes of CCSC patients treated with half-dose PDT. It is noteworthy that half-dose PDT, as a safe therapeutic method for CCSC, could potentially worsen choroidal ischemia, which is a pathophysiologic cause of neovascularization progressions in some cases, while OCTA is helpful in identifying these CC changes and following these lesions over time to better understand how they progress.

## Data Availability

The datasets used and/or analyzed during the current study are available from the corresponding author on reasonable request.

## Abbreviations

OCTA: Optical coherence tomography angiography
CC: Choriocapillaris
CCSC: Chronic central serous chorioretinopathy
SRF: Subretinal fluid
RPE: Retinal pigment epithelial
PED: Pigment epithelial detachment
PDT: Photodynamic therapy
FSV: Flow signal voids
EDI-OCT: Enhanced depth imaging optical coherence tomography
SFCT: Subfoveal choroidal thickness
ICGA: Indocyanine green angiography
CNV: Choroidal neovascularization
