# Correction to: Assessment of choriocapillary blood flow changes in response to half-dose photodynamic therapy in chronic central serous chorioretinopathy using optical coherence tomography angiography

**DOI:** 10.1186/s12886-020-01700-w

**Published:** 2020-11-12

**Authors:** Juejun Liu, Changzheng Chen, Lu Li, Yishuang Xu, Zuohuizi Yi, Lu He, Hongmei Zheng

**Affiliations:** Eye Center, Renmin Hospital of Wuhan University, Wuhan, China

**Correction to: BMC Ophthalmology 20, 402 (2020)**

**https://doi.org/10.1186/s12886-020-01674-9**

Following publication of the original article [1], we were notified that there were no colour arrow marks on Figs. 3, 4 and 5, even though these are mentioned in the figures legends and annotation. The corrected figures are shown below.

**Fig. 3:**
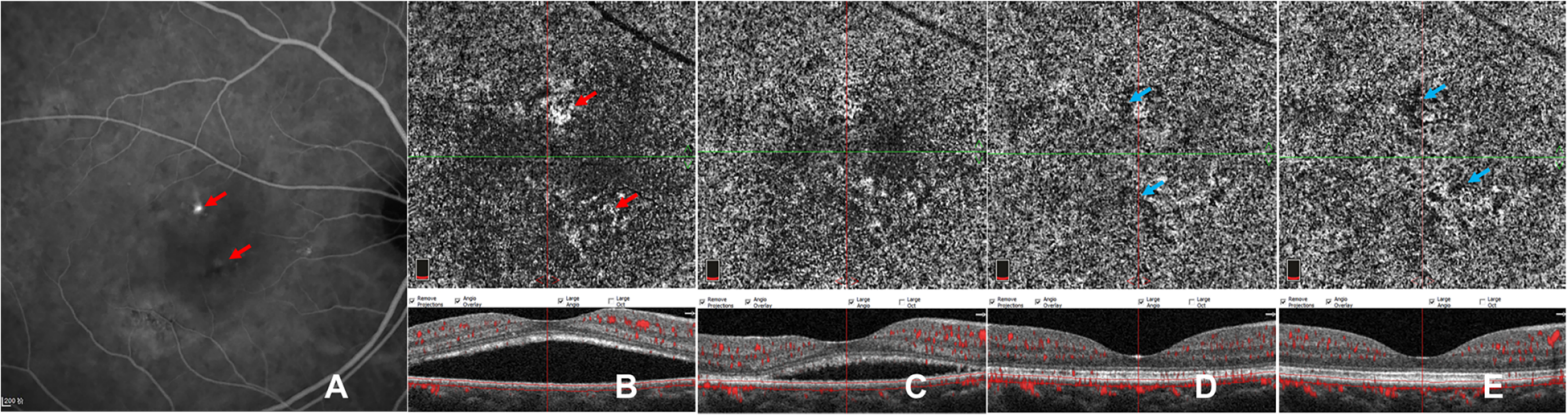
Images of a case showing CC pattern of gradually increasing flow signals after PDT. ICGA (**a**) of baseline reveals partial choriocapillary hyperpermeability (red arrow) and focal hypo-fluorescent areas (blue arrow). En-face OCTA (B-F) of CC slab and the corresponding cross-sectional B-scan OCT (**b-f**) demonstrated the CC flow changes with time. Dilatation of CC (red arrow) accompanied by dark areas (blue arrow) can be seen at baseline (**b**). Recovery sign of increasing flow signals and decreasing dark areas was found at 1 week (**c**) after half-dose PDT and at the following 1-m (**d**), 3-m (**e**) and 6-m (**f**) follow-ups, while foci of dark areas (blue arrows) remained

**Fig. 4:**
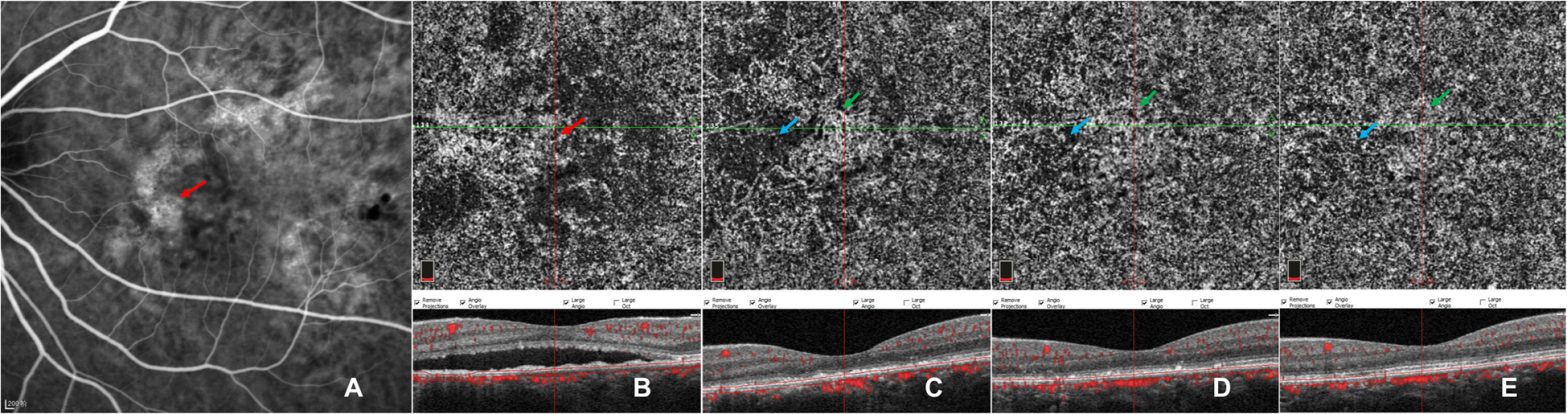
Images of a case showing CC pattern of transient network of neovascularization. ICGA (**a**) of baseline revealed widespread lesions of choriocapillary hyperpermeability (red arrow) with hypo-fluorescent areas within them. En-face OCTA (**b**-**f**) of CC slab and the corresponding cross-sectional B-scan OCT (**b-f**) demonstrated the CC flow changes with time. Local dialed CC patterns in macular region surrounded by defused flow signal void were noticeable at baseline (**b**). An emerging network of neovascularization (green arrow) accompanying foci of reduced flow signals (blue arrow) was observed at 1 week after half-dose PDT (**c**), which gradually subsided (green arrows) during subsequent follow-ups of 1 month (**d**), 3 months (**e**) and 6 months (**f**) while focally recovering with CC perfusion (blue arrow)

**Fig. 5:**
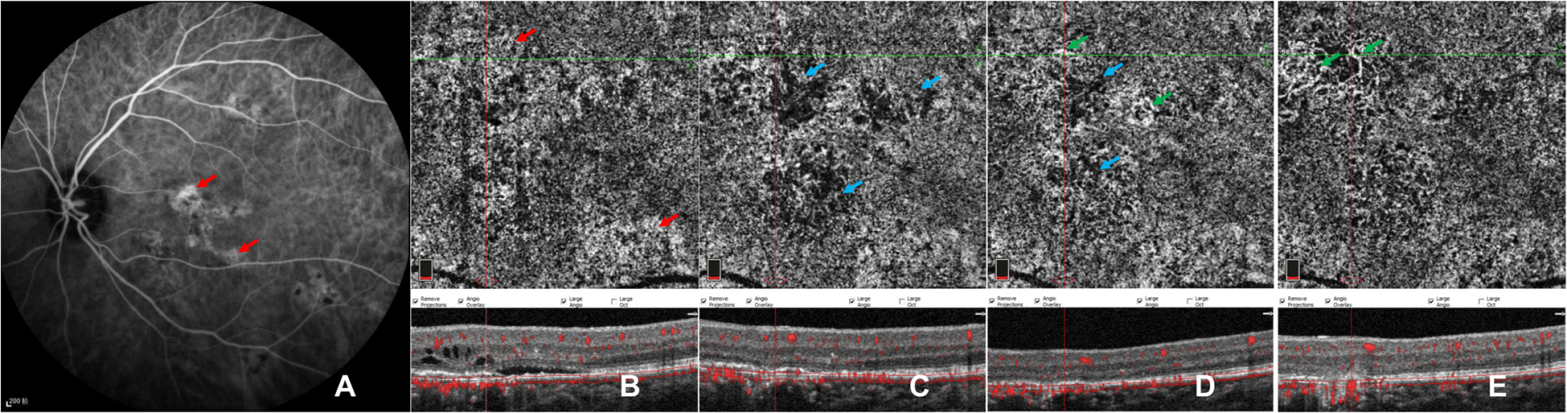
Images of a case showing CC pattern of worse CC ischemia followed by persistent type I CNV. ICGA (**a**) of baseline revealed multifocal choriocapillary hyperpermeability (red arrows). En-face OCTA (**b**-**f**) of CC slab and the corresponding cross-sectional B-scan OCT (**b-f**) demonstrated the CC flow changes with time. Defused dilatation of CC (red arrow) (**b**) can be detected at baseline, with punctate dark areas within the lesions. Local worse CC ischemia (blue arrows) was found at 1 week after half-dose PDT (**c**), combined with dynamic changes of neovascularization of sprouts (**d**) at 1-m follow-up, and grew with loose network of CNV (green arrows) during follow-ups of 3 months (**e**) and 6 months (**f**) while focally recovering with CC perfusion (blue arrow)

The original article has been corrected.
